# Chylous Mesenteric Cyst: A Rare Surgical Entity—A Case Report From Nepal

**DOI:** 10.1002/ccr3.71973

**Published:** 2026-01-29

**Authors:** Ashwini Gurung, Alisha Rai, Arbin Joshi

**Affiliations:** ^1^ Department of General Surgery and Urology B and B Hospital Lalitpur Nepal; ^2^ Department of Emergency Medicine B and B Hospital Lalitpur Nepal

**Keywords:** abdominal mass, histopathology, laparoscopic excision, lymphatic malformation, mesenteric cyst, ultrasound abdomen

## Abstract

Chylous Mesenteric Cyst is an uncommon benign abdominal cyst that can arise anywhere along the mesentery of the gastrointestinal tract, from the duodenum to the rectum. It is often asymptomatic but can rarely present with abdominal discomfort or a palpable abdominal mass. Final diagnosis typically relies on histopathology examination following laparoscopic or open excision. We present a 54 years old male who initially presented with right lower abdominal discomfort and lump. Computed Tomography (CT) demonstrated a large thin walled cystic lesion abutting and displacing the right ureter. Pre‐operative ureteric stenting and laparoscopic excision of the cyst was planned. The cyst contained 700 mL of chylous fluid and was excised in toto. Histopathological examination was consistent with chylous mesenteric cyst. The ureteric stent was removed at the end of surgery. The patient was discharged with favorable outcome in second post‐operative day. Mesenteric cysts are rare entities and appropriate planning is essential for a favorable outcome. Our case report demonstrates successful management of this rare condition with the use of minimally invasive surgery.

## Key Clinical Message

Chylous mesenteric cysts are benign abdominal cysts that can occur along the mesentery of the gastrointestinal tract. Often asymptomatic, they may present as a palpable mass. Diagnosis is confirmed through histopathology, with excision considered the gold standard. We present a rare case of mesenteric cyst successfully managed with minimally invasive surgery.

## 1 Introduction

Chylous mesenteric cysts are very unusual, with 820 cases reported to date [1, 2]. These cysts can arise from the mesentery of any part of the gastrointestinal tract, from the duodenum to the rectum [3]. The most commonly involved site is the small bowel (60%) followed by the large bowel (24%). The cyst may rarely extend into the retroperitoneum (14.5%). First identified during an autopsy of an 8‐year‐old boy by Benevieni in 1507, the term chylous mesenteric cyst was first described by Rokitansky in 1842 [4]. Compromising 7.3% of all abdominal cysts, its incidence is 105,000 in adults and 1:20,000 in children [5]. These cysts are found highest in the fourth decade of life, with a male predominance [3, 6].

Chylous mesenteric cysts are often found incidentally. A proportion of patients may present with non‐specific symptoms or with symptoms resulting from complications [2]. Diagnosis is typically made after excluding other causes of abdominal pain and surgical enucleation is the treatment of choice [3, 7]. It is important to note that approximately 3% of chylous mesenteric cysts have the potential to become malignant, the commonest being sarcoma [1, 2].

## 2 Case History/Examination

A fifty‐four‐year‐old male with diabetes and hypertension presented to our outpatient department with a painless lump on his lower right abdomen that had been gradually growing over the past three months. The mass was associated with abdominal discomfort. However, he did not exhibit other symptoms like fever or abdominal distention. His bowel habits were normal. On examination, a well‐built individual with stable vital signs and no signs of pallor and icterus was noted. His abdomen was soft with audible bowel sounds. However, a well‐defined oval‐shaped smooth and tensely cystic intra‐abdominal mass of about 10 × 10 cm was noted in the right lumbar and right iliac region. The mass was non‐tender, mobile, non‐pulsatile and did not disappear upon leg elevation. No cough impulse was detected.

## 3 Methods (Differential Diagnosis, Investigations and Treatment)

**FIGURE 1:**
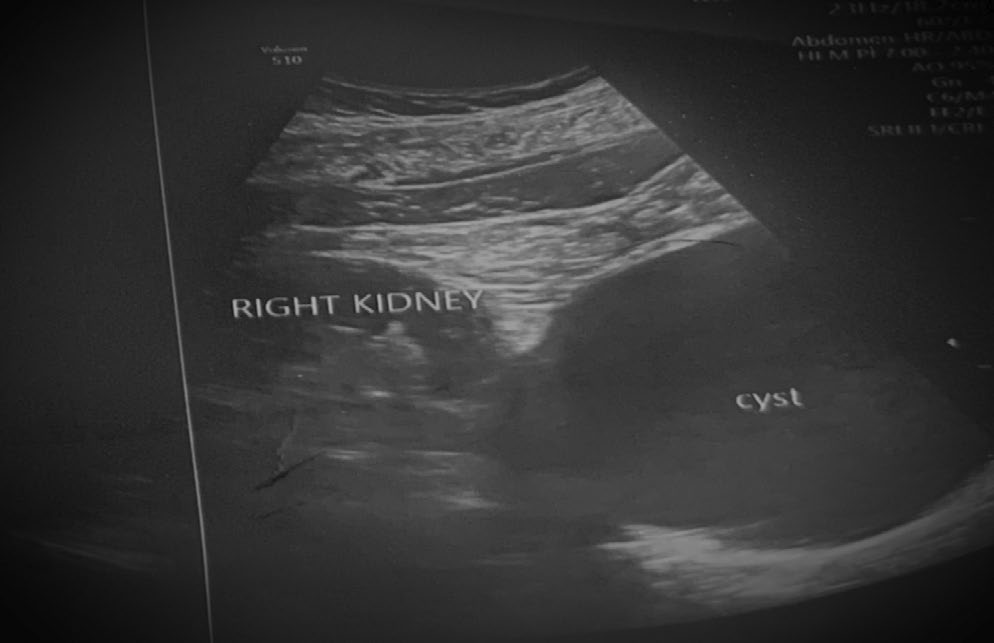
Ultrasound of abdomen showing cyst on lower pole of right kidney.

**FIGURE 2:**
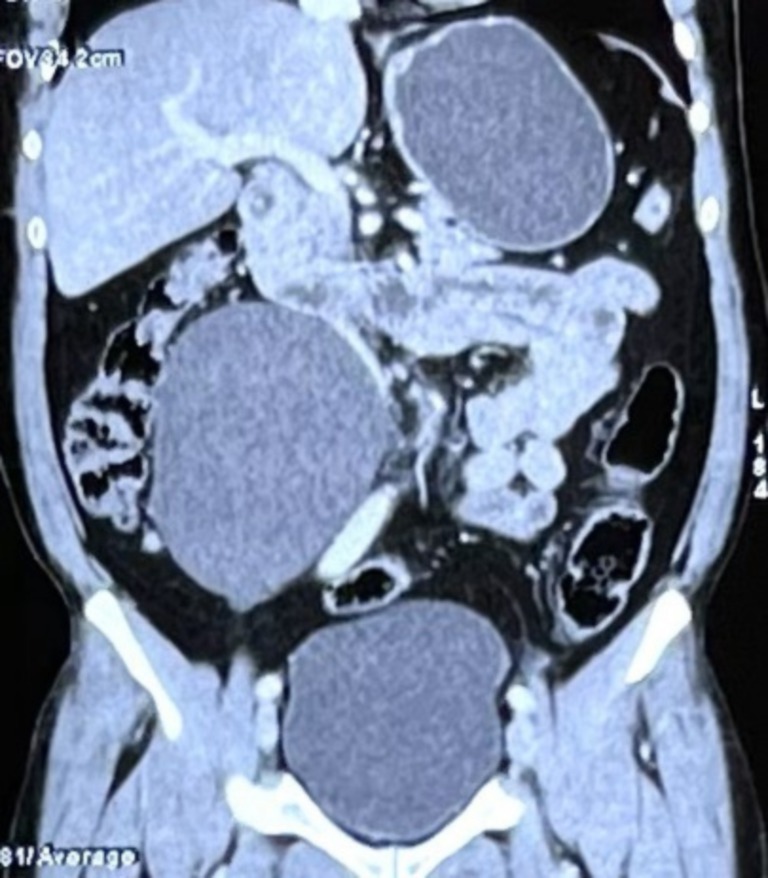
Contrast enhanced CT abdomen showing cystic lesion in the right lumbar region.

An abdominal ultrasound was done which revealed a large cystic lesion near the lower pole of the right kidney and right pelvis as shown in Figure 1, suggestive of a peritoneal cyst. Incidentally, multiple cholelithiasis in a normal wall‐thickness gall bladder was also noted. Subsequently, a contrast enhanced CT abdomen was performed which showed a large 10.4 × 10.1 × 12.5 cm non‐enhancing, thin‐walled cystic lesion in the right lumbar region with displacement of the right proximal and mid ureter as depicted in Figure 2. Based on these findings, a diagnosis of mesenteric cyst was made.

The case notes and imaging were discussed in a multi‐disciplinary meeting with urologists who recommended an intra‐operative retrograde ureterogram with or without stenting.

**FIGURE 3:**
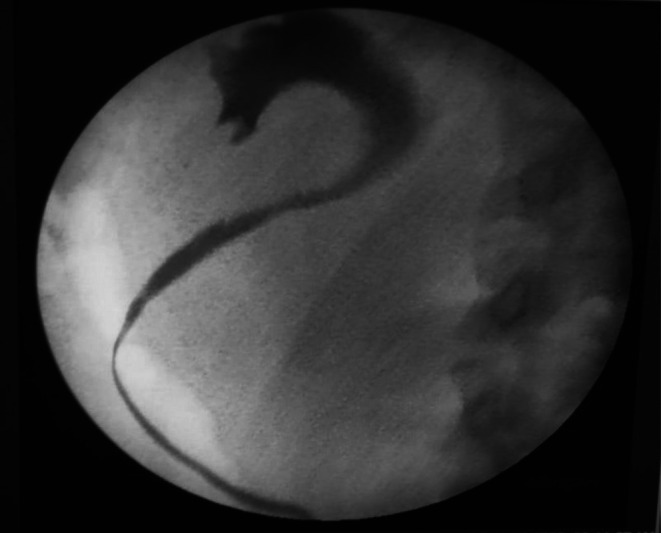
Retrograde ureterogram showing lateral displacement of right ureter and mild hydronephrosis.

Under general anesthetics, the urology team performed a retrograde ureterogram which confirmed a lateral displacement of the right ureter with mild hydroureteronephrosis as shown in Figure 3. The right ureter was stented. This was followed by the general surgeons undertaking laparoscopic excision of the cyst.

**FIGURE 4:**
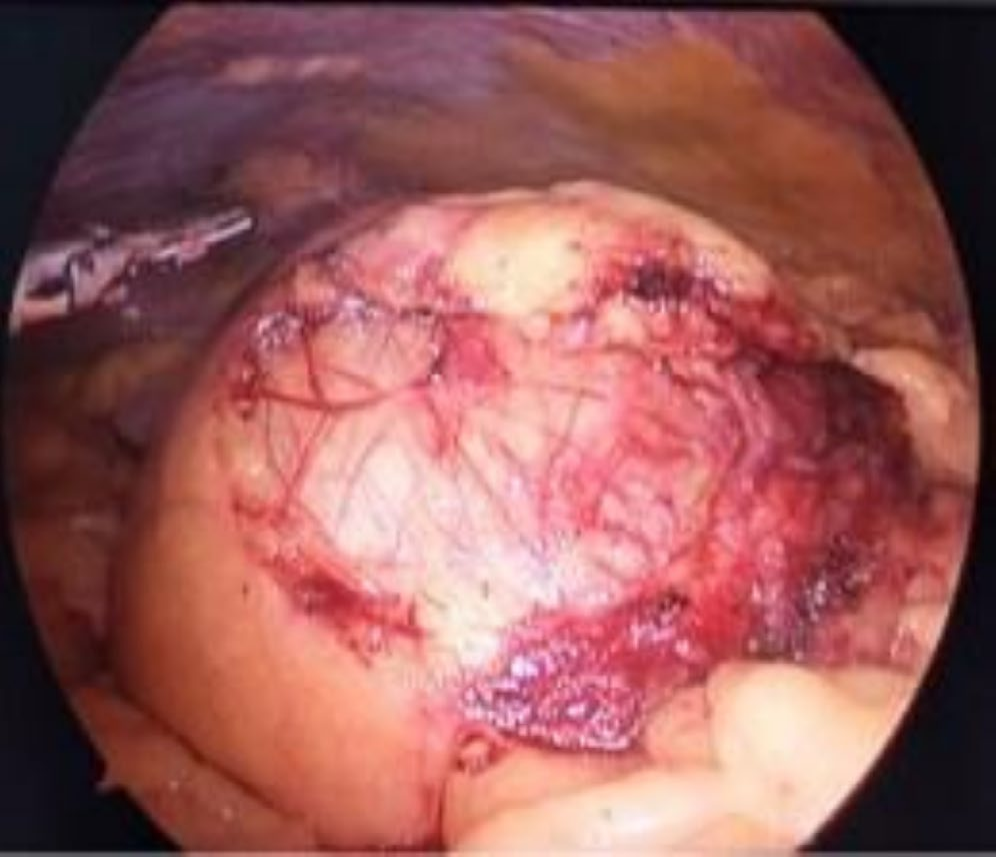
Laparoscopic view showing mass of 15 × 20 cm within the mesentery of terminal ileum. HPE showing Features of Lymphangitis.

**FIGURE 5:**
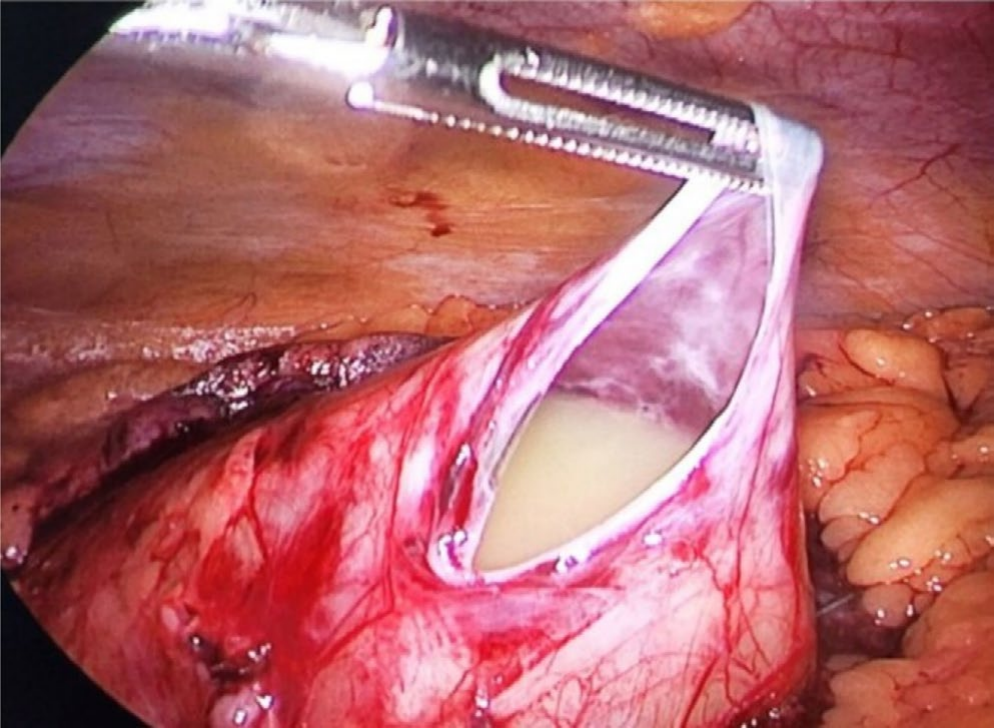
Mesenteric cyst containing chylous fluid.

Laparoscopy demonstrated a large cystic mass of approximately 15 × 20 cm size within the mesentery of terminal ileum as shown in Figure 4. The cyst was opened and approximately 700 mL of chylous fluid was drained from it as shown in Figure 5. The cyst was removed in completion preserving the vascularity of terminal ileum. At the end of the operation, the ureteric stent was removed.

## 4 Conclusion and Results (Outcome and Follow Up)

**FIGURE 6:**
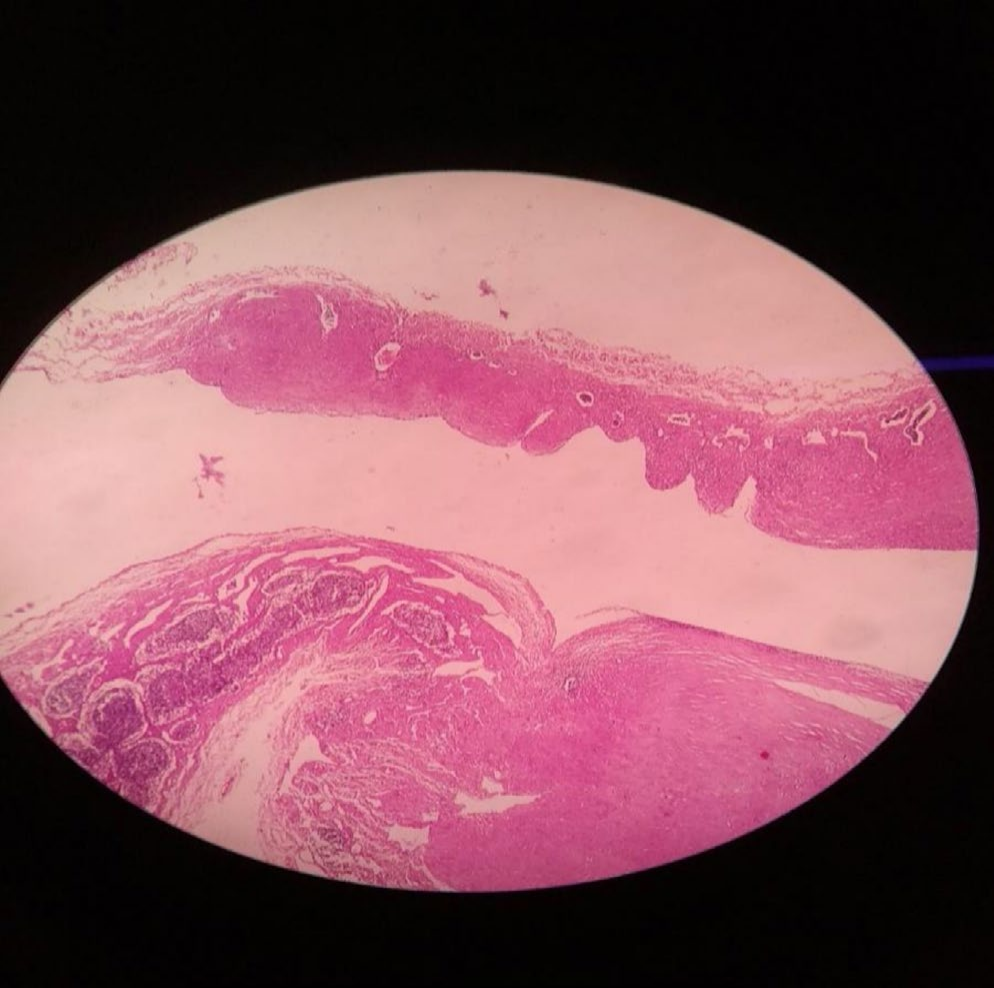
Histopathological examination showing features of lymphangitis.

Microbiological analysis had no significant findings. The histopathological examination revealed a fibro collagenous wall with flattened lining, containing lymphocytes with lymphoid follicles in the wall as well as thin‐walled vascular channels all of which were consistent with lymphangioma as presented in Figure 6. A confirmatory diagnosis of chylous mesenteric cyst was thus made.

He made an uneventful recovery and was discharged on his second postoperative day.

## 5 Discussion

Chylous mesenteric cysts are very uncommon benign intra‐abdominal cysts, first discovered during an autopsy of an 8‐year‐old boy by Italian anatomist Benevieni in 1507. The understanding and identification of these cysts have evolved over time. In 1842, Von Rokitansky first introduced the term “chylous mesenteric cyst.” Thirty‐eight years later, Tillaux pioneered the successful excision of the cyst. Since 1993, open surgical excision has transitioned to laparoscopic techniques [4, 8]. Due to their rarity, studies on chylous mesenteric cysts are limited.

These cysts arise from the mesentery of either the small or large bowel. In one of the largest studies conducted on 162 individuals by Kurtz et al., more than half of the cysts were found to originate from the small bowel, specifically the terminal ileum, which is consistent with our findings. In rare instances, these cysts can extend up to the retroperitoneum like in our case [4].

The reason for this cyst to occur is still not known. However, different hypotheses have been proposed. The most famous theory is that proposed by Gross, which posits that cyst formation arises from lymphatic cells that fail to communicate with the rest of the lymphatic system [9]. Other theories suggest that risk factors like infection, trauma, neoplasia, and pelvis surgery may block the lymphatic drainage, resulting in cyst formation [10].

The histopathological appearance can also classify the cyst into six distinct groups based on their origin:

1. Lymphatic cyst: Simple lymphatic cyst and lymphangioma
2. Mesothelial cyst: Simple mesothelial cyst, benign cystic mesothelioma and malignant cystic mesothelioma
3. Enteric cyst: Enteric cyst and intestinal duplication cyst
4. Urogenital cyst
5. Mature cystic teratoma: Dermoid cyst
6. Non‐pancreatic pseudocyst: Traumatic or infectious origin

Among these, the most prevalent one is lymphatic in origin [11].

These cysts can vary significantly in size, number, and content. Size can range from as small as 2 cm to as big as 36 cm, potentially occupying the entire abdominal cavity [4]. They can be single or multiple, and unilocular or multilocular [12].

Microscopically, cysts are identified by walls lined with a single layer of fibrous tissue or endothelial cells, with or without calcification [2]. In our case, the cyst was single, approximately 10 cm big and of mesothelial origin.

The symptoms of these cysts depend on various factors, including their size, location, number, and any complications [7]. They may be asymptomatic and discovered incidentally, or present with non‐specific symptoms or acute abdomen. Abdominal pain is reported in 55%–82% of cases, while 55%–61% may present with a mass as in our case. Abdominal distention can occur in 17%–61% cases [2]. Rarely, a large cyst can compress the nearby structures such as bowel, leading to features of bowel obstruction and volvulus. Volvulus may eventually cause ischemia, gangrene, and perforation leading to peritonitis [2]. In our patient, the cyst displaced the ureter laterally, causing hydroureteronephrosis, though he remained asymptomatic.

Presentation in children is different from adults, with the child being more prone to acute abdomen [3]. In cases of trauma, cysts can rupture and bleed.

Our patient presented with a gradually progressive abdominal mass accompanied by discomfort, which is the second most common clinical presentation [2]. These non‐specific symptoms make diagnosing a mesenteric cyst challenging, as they can mimic conditions such as pancreatic pseudocysts, cystic tumors, aortic aneurysms, and other causes of abdominal pain [13].

Historically, diagnosing a mesenteric cyst was based on exclusion [4]. However, with advancements in radiology, ultrasound of abdomen and CT scans has gained more popularity. Ultrasound can determine the cyst's size, shape, content, and wall thickness. CT scans further aid in accurately identifying the cyst's origin and its relationship to other abdominal structures, which is crucial for surgical planning [14, 15]. Diagnostic laparoscopy is the mainstay of the diagnosis along with histopathological examination [16].

In our case, both ultrasound and CT scans were performed, enabling a preoperative diagnosis that was later confirmed by histopathological examination.

The primary treatment is enucleation of the cyst either open or laparoscopically [3, 10, 17, 18]. Various other approaches like marsupialization, drainage, percutaneous drainage, sclerotherapy, and deroofing have been described. However, enucleation is preferred over any other treatment modalities as the rest of the modalities have high chances of recurrence and infection [2, 3, 7, 15]. Therefore, the gold standard treatment for mesenteric cyst is complete surgical excision of the cyst unless there are adhesions or the cyst shares a common blood supply with an intestinal segment. In such cases, complete removal of the cyst with segmental resection of the intestine is required [12, 19].

## 6 Conclusions

Mesenteric cysts are a rare clinical entity, accounting for only 7.3% of all abdominal cysts. Their etiology remains unclear, and clinical presentations are often either non‐specific or asymptomatic, making diagnosis challenging for physicians. Despite their rarity, mesenteric cysts should be considered in differential diagnoses, and appropriate imaging modalities should be utilized. Surgical enucleation is the preferred treatment to prevent recurrence.

## Author Contributions

**Ashwini Gurung:** conceptualization, data curation, formal analysis, resources, writing – original draft. **Alisha Rai:** investigation, methodology, project administration, software, writing – original draft. **Arbin Joshi:** supervision, validation, writing – review and editing.

## Funding

The authors have nothing to report.

## Consent

Both verbal and written consent was obtained from the patient to publish this report. A copy of the written consent form is available for review on request.

## Conflicts of Interest

The authors declare no conflicts of interest.

## Data Availability

The authors have nothing to report.
